# The Association of Lifestyle Patterns with Prediabetes in Adults from Families at High Risk for Type 2 Diabetes in Europe: The Feel4Diabetes Study

**DOI:** 10.3390/nu15143155

**Published:** 2023-07-14

**Authors:** Niki Mourouti, Christina Mavrogianni, Theodora Mouratidou, Stavros Liatis, Päivi Valve, Imre Rurik, Péter Torzsa, Greet Cardon, Yulia Bazdarska, Violeta Iotova, Luis A. Moreno, Konstantinos Makrilakis, Yannis Manios

**Affiliations:** 1 Department of Nutrition and Dietetics, Hellenic Mediterranean University, 72300 Sitia, Greece; nmourouti@hua.gr (N.M.); tmouratidou@hmu.gr (T.M.); 2 Institute of Agri-Food and Life Sciences, Hellenic Mediterranean University Research Centre (Agro-Health), 71410 Heraklion, Greece; 3 Department of Nutrition and Dietetics, School of Health Science and Education, Harokopio University, 17671 Athens, Greece; cmavrog@hua.gr; 4 First Department of Propaedeutic Medicine, Laiko General Hospital, Medical School, National and Kapodistrian University of Athens, 11527 Athens, Greece; sliatis@med.uoa.gr (S.L.); kmakrila@med.uoa.gr (K.M.); 5 Department of Public Health and Welfare, Finnish Institute of Health and Welfare, FI-00271 Helsinki, Finland; paivi.valve@thl.fi; 6 Department of Family Medicine, Semmelweis University, 1085 Budapest, Hungary; rurik.imre@med.unideb.hu (I.R.); torzsa.peter@med.semmelweis-univ.hu (P.T.); 7 Department of Movement and Sports Sciences, Faculty of Medicine and Health Sciences, Ghent University, 9000 Ghent, Belgium; greet.cardon@ugent.be; 8 Department of Pediatrics, Medical University Varna, 9002 Varna, Bulgaria; yuliya.bazdarska@mu-varna.bg (Y.B.); iotova_v@yahoo.com (V.I.); 9 Growth, Exercise, Nutrition and Development (GENUD) Research Group, University of Zaragoza, 50009 Zaragoza, Spain; lmoreno@unizar.es

**Keywords:** prediabetes, lifestyle patterns, principal component analysis

## Abstract

The increasing prevalence of prediabetes globally does not bode well for the growing epidemic of type 2 diabetes (T2D) and its complications. Yet there is a lack of studies regarding lifestyle patterns (LPs) and their association with prediabetes. The present study aimed to examine the association of different LPs with the existence of prediabetes in adults from families at high risk for T2D in Europe. In total, 2759 adults (66.3% females) from six European countries were included in this cross-sectional analysis using data from the baseline assessment of the Feel4Diabetes study. Anthropometric, sociodemographic, dietary and behavioral data were assessed, and fasting blood glucose measurements were also obtained. LPs were derived via principal component analysis. Two LPs were derived, explaining 32% of the total variation. LP 1 was characterized by breakfast consumption, high consumption of fruits and berries, vegetables and nuts and seeds, and low consumption of salty snacks and soft drinks with sugar, while LP 2 was characterized by high consumption of salty and sweet snacks, soft drinks with sugar and juice with sugar and sedentary behavior. After adjusting for various confounders, LP 2 was positively associated with the existence of prediabetes (odds ratio = 1.02, 95% CI 1.01–1.04), while LP 1 was not significantly associated with prediabetes. Understanding LPs would provide necessary evidence for planning intervention and education strategies for prediabetes and T2D.

## 1. Introduction

Prediabetes is increasingly recognized as an important metabolic state; it is typically an umbrella term encompassing impaired fasting glucose (IFG) and/or impaired glucose tolerance (IGT) [1]. However, comprehensive global prevalence data on prediabetes are lacking. In 2021, the International Diabetes Federation (IDF) estimated the global IGT prevalence at 10.6% in both men and women [2]. The latter estimate corresponds to approximately 541 million adults aged 18–99 years, and in 2045, the prevalence of IGT is expected to increase in young adults (aged 45 years or younger) and the very old (aged 70 years or older) and slightly decrease among middle-aged adults (aged 45–69 years). [2]. The vast majority of individuals with prediabetes (72.2%) reside in low- and middle-income countries (LMICs); the North American and Caribbean regions have the highest IGT prevalence (13.8%), and the European region has the lowest prevalence (5.1%) [2]. The increasing prevalence of prediabetes globally is a major public health concern and does not bode well for the growing epidemic of diabetes and its complications. The natural history of the condition is well documented, its detection can be straightforward, and evidence for its effective treatment has accumulated over the past two decades [1]. However, there is controversy regarding the optimal definition of prediabetes, and active recognition and treatment of prediabetes have lagged, as clinicians may fail to see it as a disease state that needs addressing.

Prediabetes is a complex metabolic state with various risk factors which are the same as those for type 2 diabetes (T2D), including family history, existence of other chronic diseases (i.e., hypertension and dyslipidemia) and behavioral risk factors. Overweight/obesity, diet and physical inactivity are the most dominant. Intervention strategies applied to prediabetic subjects range from specific foods to combined diets incorporating an amalgamation of food types. In their majority, specific food/food groups including grains, plant-based foods and nuts [3,4,5,6,7,8,9,10,11] as well as specific diets (i.e., low-calorie and low-glycemic-index diet) [12,13,14,15,16,17] seem to have a protective role in the onset of prediabetes. Moreover, many studies have indicated that a combination of diet and exercise has demonstrated better results than each of these alone [18,19], highlighting the need for more lifestyle alterations.

However, it should be noted that the majority of previous studies followed the approach of assessing single nutrients or food items, instead of assessing patterns. Nevertheless, it is accepted that people do not eat isolated nutrients, but they consume meals consisting of a variety of foods with complex combinations of micro- and macronutrients. Moreover, food-specific analyses in relation to the characteristics of people or incidence of a disease share many methodological limitations (i.e., high collinearity of the food variables, inability to test for the synergistic effects of foods, etc.). Thus, it has been at least two decades since several investigators in the field of nutritional epidemiology have suggested using a holistic approach when exploring its impact on disease prevention, giving much attention to pattern analysis [20,21]. Compared to the classical methods used in nutritional epidemiology, the approach of the lifestyle pattern confers a holistic representation in investigating the predisposing factors for the emergence of non-communicable chronic diseases (NCDs) [22]. Instead of examining a single factor (e.g., diet, physical activity, smoking, alcohol consumption and sleep) and its association with health/illness, this approach studies the entire lifestyle pattern and the interrelationships that may exist between these various lifestyle factors. As a result, a lifestyle pattern is distinguished as a dynamic interaction between factors, rather than emphasizing each individual factor.

Yet there is a lack of studies regarding lifestyle patterns and their association with prediabetes. However, individuals with prediabetes are at risk for future T2D, while prediabetes is also associated with a high burden of cardiometabolic risk factors and poor outcomes. Thus, the effects of a lifestyle pattern on health would outweigh those of its components taken individually (diet, physical activity, etc.) and could thus detect more associations and implications in real life. Therefore, following the holistic approach, the present work aimed to examine the possible association of lifestyle patterns with prediabetes in adults from families at high risk of T2D in Europe.

## 2. Materials and Methods

### 2.1. Study’s Design

This cross-sectional study was a secondary analysis of baseline data of high-risk families participating in the large pan-European population-based cohort, the Feel4Diabetes Study (Families across Europe following a healthy Lifestyle for Diabetes prevention). Feel4Diabetes was a large school- and community-based intervention among families from vulnerable groups in six European countries, undertaken from 2016 to 2018 (National Clinical Trial number, NCT02393872; https://feel4diabetes-study.eu/, accessed on 15 June 2023). The aim of the intervention was to promote a supportive social and physical environment in home and school settings to assist families in adopting a healthy and active lifestyle. In Bulgaria and Hungary (i.e., LMICs), all families were considered vulnerable and eligible to participate in the study, while in Belgium, Finland, Greece and Spain (i.e., high income countries-HICs), families from municipalities with the lowest educational level or the highest unemployment rate (as retrieved from official resources and authorities) were included as vulnerable groups.

During the first-stage screening, in each country, primary schools located in the selected “vulnerable” areas were used as the entry point to the community. Children attending the first three grades of compulsory education as well as their parents and grandparents (wherever feasible) were recruited to the study. Of these recruited families, the “high-risk families” were identified based on T2D risk estimation, using the Finnish Diabetes Risk Score (FINDRISC) questionnaire. A family was regarded as “high-risk” if at least one parent fulfilled the country-specific cut-off point for FINDRISC that indicated increased T2D risk (for the majority of countries, considering the young age of the participants, that was set as a FINDRISC score ≥ 9). Self-administrated FINDRISC questionnaires were collected from 11,396 families, and then, all the parents and/or grandparents of the ‘‘high-risk families”, irrespectively of their individually calculated FINDRISC, were invited to undergo a more detailed assessment (second screening) delivered in local community centers or during home visits (in Belgium). From the identified ‘‘high-risk families”, 3148 parents from 2535 families underwent the second screening. A detailed description of methods has been previously published [23,24].

### 2.2. Bioethics

The Feel4Diabetes study adhered to the Declaration of Helsinki and the conventions of the Council of Europe on human rights and biomedicine [23]. All participating countries obtained ethical clearance from the relevant ethical committees and local authorities. More specifically, in Belgium, the study was approved by the Medical Ethics Committee of the Ghent University Hospital (ethical approval code: B670201524437); in Bulgaria, by the Ethics Committee of the Medical University of Varna (ethical approval code: 52/10-3-2016r) and the Municipalities of Sofia and Varna, as well as the Ministry of Education and Science local representatives; in Finland, by the hospital district of Southwest Finland ethical committee (ethical approval code: 174/1801/2015); in Greece, by the Bioethics Committee of Harokopio University (ethical approval code: 46/3-4-2015) and the Greek Ministry of Education; in Hungary, by the National Committee for Scientific Research in Medicine (ethical approval code: 20095/2016/EKU); and in Spain, by the Clinical Research Ethics Committee and the Department of Consumers’ Health of the Government of Aragón (ethical approval code: CP03/2016). All participants gave their written informed consent prior to their enrolment in the study.

### 2.3. Study Population

From the 3148 parents who underwent the second screening, 106 (3.4%) were excluded from the current analysis because they were new cases of diabetes type 2, and 283 (9%) participants because they were under any treatment for prediabetes and/or diabetes. As a result, the total sample of the present study consisted of 2759 adults from the “high-risk families”, including individuals with normal fasting plasma glucose (FPG) levels, undiagnosed prediabetes and who were not under any treatment for prediabetes and/or diabetes.

### 2.4. Anthropometry

For the weight measurement, the participants had to wear light clothing and remove the shoes, while for the height measurement, they had to stand in an erect position without shoes, shoulders relaxed, arms by the side and head aligned in the Frankfort plane. Weight was recorded to the nearest 0.1 kg using a calibrated SECA digital scale (SECA 813, Hamburg Germany), and height was recorded to the nearest tenth of a centimeter (i.e., 0.1 cm) using a telescopic stadiometer (SECA 213). All volunteers were categorized by the body mass index (BMI) cut-off points. BMI was calculated by the formula [weight/height^2^]. Waist circumference (WC) was measured midway between the lowest rib margin and the iliac crest to the nearest 0.1 cm using a non-elastic measuring tape (SECA 201). BMI and WC were classified based on the World Health Organization (WHO) criteria [25].

### 2.5. Blood Indices

Blood tests were performed on the same day with the anthropometric measurements by professional staff on all participants in the morning (8:30–10:30) after 12 h overnight fasting. Measurements of FPG were acquired. Blood samples directed for glucose measurement were collected in tubes with sodium fluoride (10.0 mg) and potassium oxalate (8.0 mg) for the inhibition of glycolysis. Participants were classified according to the American Diabetes Association (ADA) criteria in the following categories: normoglycemic (FPG < 100 mg/dL; 5.6 mmol/L), prediabetics (FPG 100–125 mg/dL; 5.6–6.9 mmol/L) and having T2D (FPG > 126 mg/dL; 7.0 mmol/L) [26]. Measurements of serum total and high-density lipoprotein (HDL) cholesterol and triglyceride (TG) levels were also acquired. Low-density lipoprotein (LDL) cholesterol was calculated using the Friedewald formula [27]. Dyslipidemia was defined as total cholesterol (TC) ≥ 200 mg/dL, TG ≥ 150 mg/dL, LDL-C ≥ 130 mg/dL, or HDL-C < 40 mg/dL (for men) and HDL-C < 50 mg/dL (for women) [28].

### 2.6. Blood Pressure Measurement

Blood pressure was measured on the right arm, in a sitting position using electronic sphygmomanometers (OMRON M6 or OMRON M6 AC) after five minutes of rest, on three occasions, at one-minute intervals. The measurements were conducted in a private, quiet place with proper temperature. The existence of hypertension (HTN) was based on elevated systolic blood pressure (SBP), diastolic blood pressure (DBP) or both according to the latest European guidelines [29].

### 2.7. Dietary Assessment

Dietary information was derived from adults using a questionnaire measuring the frequency of meals and snacks, the frequency and quality of consumption of certain types of food at breakfast, and the reasons for skipping breakfast. Moreover, participants were asked to record the quantity (i.e., in cups or portions per day or week), quality (white or whole wheat products, type of meat, type of snacks) and frequency of consumption of particular types of food and beverages (dairy products, bread, fats, fruits and berries, vegetables, legumes, red meat, processed meat, white meat, fish, seafood, salty snacks, sweet snacks, nuts and seeds, water, tea, coffee, alcoholic beverages, sugar or non-sugar sweetened soft drinks). Regarding frequency, the answer options were <1, 1–2, 3–4, 5–6 or 7 times per week [30,31].

### 2.8. Demographic and Behavioral Characteristics

Standardized self-reported questionnaires (translated into each local language) were used for all study participants to gather information on basic sociodemographic characteristics (age, ethnicity, education level, marital status, occupation) along with information concerning smoking, physical activity, sedentary behaviors (i.e., sitting hours, screen time) and sleep duration as well as their determinants.

### 2.9. Statistical Analysis

Continuous variables were checked for normality using the Kolmogorov–Smirnov test and are presented as median and interquartile range (IQR, 25th–75th percentile), while categorical variables are presented as frequencies. Kruskal–Wallis test for independent samples was used to evaluate mean differences of the continuous variables due to non-normality of the data. Associations between categorical variables were tested by the calculation of the chi-square test.

To obtain lifestyle patterns, the factor analysis with the principal components method (PCA) was applied [32]. Dietary intake of specific food groups/beverages (i.e., fruits and berries, vegetables, legumes, salty snacks, sweet snacks, soft drinks with sugar, juice with sugar, nuts and seeds and alcohol) and lifestyle habits (i.e., breakfast consumption, sedentary behavior) were included in the analysis. The correlation matrix of the variables used showed that there were several correlation coefficients with absolute value >0.3; moreover, the *phi* coefficient (another measure of the inter-relationship of variables) was 0.61, and the Kaiser–Meier–Oklin criterion was 0.62 (which suggests very good inter-correlation). Therefore, the factor analysis would be effective for assessing meaningful lifestyle patterns. An orthogonal rotation (*rotate* with *varimax* option) was used to derive optimal non-correlated components (i.e., lifestyle patterns). A correlation matrix was used for the extraction of the components. The information was rotated in order to increase the representation of each variable as a component [32]. According to the criterion proposed by Kaiser, i.e., the number of components that should be retained is equal to the number of eigenvalues that are greater than 1, since these components explain more information than the individual variables; it was also concluded that the first two components should be extracted here. Based on the principle that the component scores (loadings) are interpreted similarly to correlation coefficients [32] and thus higher absolute values indicate that the variable contributes most to the construction of the component, the components (patterns) were named according to scores of the variables that were >0.3.

Furthermore, multiple logistic regression analysis was applied to evaluate the association of the lifestyle patterns derived with the existence of prediabetes. The analysis accounted for the potential confounding effect of the following characteristics: age, sex, education (measured in years) as a proxy of social status, smoking status (never smoked/former smoker/current smoker) and waist circumference (measured in cm). Three different models were applied for each lifestyle pattern. Model 1: adjusted for age and sex; Model 2: age, sex, education level, smoking; Model 3: age, sex, education level, smoking, waist circumference. The results are presented as odds ratios (ORs) and their corresponding 95% confidence intervals (95% CIs). All reported *p*-values were based on two-sided tests. Statistical calculations were carried out using SPSS 25 software (SPSS Inc., Chicago, IL, USA).

## 3. Results

### 3.1. Characteristics of the Participants

In Table 1, the basic characteristics of the 2759 participants by country category are presented. The median age of the participants was 41 (IQR: 38–45), and most of them were women (66.3%). One-quarter of participants had less than 12 years of education (25.6%) and were current smokers (25.6%), with these percentages being statistically significantly lower in HICs compared with other country categories (*p* < 0.001). Moreover, almost one-quarter of participants (24%) had undiagnosed prediabetes, with these percentages being statistically significantly higher in HICs compared with LMICs (29.1% vs. 13.8%) (*p* < 0.001). With regard to weight status, the median BMI was 28 (IQR: 25–32). Regarding LMICs, participants had statistically significantly lower waist circumference and FPG than HICs (*p* < 0.001). Finally, participants were more likely to consume breakfast 4 days per week and spend at least 5 h per day sitting (Table 1).

### 3.2. Lifestyle Patterns

As described above, based on the PCA, the two first components that explained 32% of the total variation were studied here. The loadings for the two components (patterns) that represent the correlation of each lifestyle variable with the corresponding component are presented in Table 2 (in bold are the coefficients with absolute loadings >0.3, meaning those that are better correlated with the component). Since the higher absolute values indicate that the lifestyle variable contributes more to the characterization of the component [32], it could be suggested that the extracted components are characterized as follows: (a) a lifestyle pattern (component 1), which loaded heavily on breakfast consumption, high consumption of fruits and berries, vegetables and nuts and seeds, and low consumption of salty snacks and soft drinks with sugar, and (b) a lifestyle pattern that is mainly characterized by the consumption of salty snacks, sweet snacks, soft drinks with sugar and juice with sugar and sedentary behavior. Component 1 was the most dominant food pattern and explained 17.4% of the total variance, while the second component explained 14% of the total variance. Regarding the other nine components that explained the rest of the variation in food/beverage consumption and lifestyle behaviors, none of them was characterized by a specific pattern.

### 3.3. Lifestyle Patterns and Prediabetes

Three multiple logistic regression models were estimated in order to evaluate the association between the extracted lifestyle patterns and the existence of prediabetes (Table 3). The use of these models assisted in better exploring the potential effect of various confounders in the investigated relationship. The first model included age, sex and the two lifestyle patterns derived from the principal component analysis. It was observed that the lifestyle pattern that was mainly characterized by the consumption of salty snacks, sweet snacks, soft drinks with sugar and juice with sugar and sedentary behavior (component 2) was positively associated with the existence of prediabetes (*p* = 0.02), while the lifestyle pattern that represented breakfast consumption, high consumption of fruits and berries, vegetables and nuts and seeds, and low consumption of salty snacks and soft drinks with sugar (component 1) was not significantly associated with the existence of prediabetes (*p* = 0.9). In the second model, education (measured in years) as a proxy of social status and smoking status (never smoked/former smoker/current smoker) were also entered. The lifestyle pattern that was mainly characterized by the consumption of salty snacks, sweet snacks, soft drinks with sugar and juice with sugar and sedentary behavior (component 2) was positively associated with the existence of prediabetes (*p* < 0.001). The lifestyle pattern that represented breakfast consumption, high consumption of fruits and berries, vegetables and nuts and seeds, and low consumption of salty snacks and soft drinks with sugar (component 1) was not significantly associated with the existence of prediabetes (*p* = 0.6). In the third model, waist circumference (measured in cm), was included. The detrimental effect of the lifestyle pattern that was mainly characterized by the consumption of salty snacks, sweet snacks, soft drinks with sugar and juice with sugar and sedentary behavior (component 2) remained unaltered (*p* = 0.006), while the lifestyle pattern that represented breakfast consumption, high consumption of fruits and berries, vegetables and nuts and seeds, and low consumption of salty snacks and soft drinks with sugar (component 1) was again not significantly associated with the disease (*p* = 0.1). After adjusting additionally for the existence of other comorbidities (hypertension and dyslipidemia) the detrimental effect of the lifestyle pattern that was mainly characterized by the consumption of salty snacks, sweet snacks, soft drinks with sugar and juice with sugar and sedentary behavior (component 2) remained unaltered (OR = 1.02, 95% CI [1.004–1.04], *p* = 0.02) while the lifestyle pattern that represented breakfast consumption, high consumption of fruits and berries, vegetables and nuts and seeds, and low consumption of salty snacks and soft drinks with sugar (component 1) was again not significantly associated with the disease (OR = 0.99, 95% CI [0.98–1.31], *p* = 0.08). Similar results were found using BMI instead of waist circumference as a covariate.

### 3.4. Profile Analysis of the Associations of Lifestyle Patterns and Prediabetes

Multi-adjusting cannot entirely exclude residual confounding, so sub-group analysis by obesity and sex was applied. Concerning obesity, the results were similar to the ones presented above; particularly, among overweight/obese participants (i.e., BMI > 25 kg/m^2^) the lifestyle pattern that was mainly characterized by the consumption of salty snacks, sweet snacks, soft drinks with sugar and juice with sugar and sedentary behavior was positively associated with the existence of prediabetes (*p* = 0.04) after adjusting for age, sex, years of education and smoking status, while among normal-weight participants (i.e., BMI < 25 kg/m^2^), there was no significant association (*p* = 0.13). Stratified analysis by sex revealed no significant associations between the two lifestyle patterns and the existence of prediabetes for both females and males (*p* > 0.05) after adjusting for age, years of education, smoking status and waist circumference.

## 4. Discussion

In this work, the existence of prediabetes was tested in relation to lifestyle patterns extracted from the data of 2759 adults from families at risk of T2D in Europe. The analysis revealed that the lifestyle pattern which was mainly characterized by the consumption of salty snacks, sweet snacks, soft drinks with sugar and juice with sugar and sedentary behavior was positively associated with the existence of prediabetes, while the lifestyle pattern that represented breakfast consumption, high consumption of fruits and berries, vegetables and nuts and seeds, and low consumption of salty snacks and soft drinks with sugar was not significantly associated with the existence of prediabetes.

Some other investigators have evaluated patterns with only dietary data in relation to prediabetes using similar multivariate techniques and describing similar food patterns. For example, in a recent cross-sectional study conducted among 1305 participants with different glucose tolerance statuses, Pestoni et al. [33], using PCA, concluded that a Western dietary pattern characterized by high consumption of red and processed meat, alcoholic beverages, refined grains and sugar-sweetened beverages was associated with prediabetes, which is in line with the present findings. Furthermore, Bagheri et al. [34] used a semi-quantitative FFQ to assess dietary intake in 150 prediabetic subjects and 150 healthy controls. After using PCA, two dietary patterns were identified: the vegetables, fruits and legumes (VFL) dietary pattern and the sweet, solid fat, meat and mayonnaise (SSMM) dietary pattern. After adjusting for several confounding factors, the VFL dietary pattern was found to be negatively associated with prediabetes, while the SSMM dietary pattern was positively associated with prediabetes. As far as sugar-sweetened beverage (SSB) consumption is concerned, in a cross-sectional analysis among 4771 US Hispanic/Latino adults with prediabetes, high sugar-sweetened beverage (SSB) consumption was associated with increased odds of prediabetes [35]. Moreover, prospective data indicate that daily consumption of sugar-sweetened beverages has been shown to increase the risk of developing prediabetes among middle-aged adults by 46% [36].

Individuals with prediabetes have a high risk of progression to diabetes and an elevated risk of cardiovascular disease (CVD). Breakfast is commonly considered to be the most important meal of the day although many people skip it. Based on current findings, skipping breakfast can be a risk factor for impaired glucose metabolism, leading to prediabetes [37]. Furthermore, a systematic review and meta-analysis of prospective cohort studies based on 96,175 participants and 4935 cases indicated that breakfast skipping is associated with an increased risk of T2D [38]. A recent meta-analysis of cohort studies concerning a total of 221,732 participants concluded that breakfast skipping increases the risk of cardiovascular disease as well as all-cause mortality [39].

As far as sedentary behaviors and physical activity are concerned, in a recent observational prospective population-based cohort study, the Maastricht study, researchers indicated that the association between sedentary time and biomarkers of endothelial dysfunction (important in the pathogenesis of CVDs) was consistently stronger in prediabetes and T2D as compared with normal glucose metabolism status [40]. Furthermore, in a prospective, population-based cohort study, 360,047 participants enrolled in the UK Biobank were followed up for 45 non-communicable diseases (NCDs) according to ICD-10 codes. Participants who reported higher sedentary time had higher risks of 12 of 45 NCDs, including T2D and ischemic heart disease [41]. A recent systematic review and meta-analysis of 148 randomized controlled trials (RCTs) and 36 longitudinal studies indicated that long-term sedentary behavior increases the risk of CVDs in healthy adults, whereas physical activity reduces the risk of CVDs and improves indicators associated with CVDs [42]. The above findings are in agreement with the most recently published physical activity guidelines, which indicate that sedentary behavior is associated with detrimental effects on both T2D and the incidence of cardiovascular disease [43].

The present study used the holistic approach and analyzed the association of patterns with the existence of prediabetes in adults from families at high risk for T2D across Europe. The large study sample, the standardized protocols and procedures followed across all centers, and the objectively collected data (i.e., blood and anthropometric indices) safeguard the objectivity and reliability of the assessment and increase the generalizability of the findings. However, our study should be viewed in the light of some limitations. The cross-sectional nature of the study design hinders the establishment of a causal link between the specific patterns and adult prediabetes. Additionally, some of the collected data were self-reported and thus are prone to recall bias and social desirability.

## 5. Conclusions

Pattern analysis revealed behaviors in participants’ lifestyles that could not be assessed with any other method. It was found that a lifestyle pattern that is mainly characterized by high consumption of salty and sweet snacks, soft drinks with sugar and juice with sugar and sedentary behavior was positively associated with the existence of prediabetes. Understanding lifestyle patterns would provide necessary evidence for planning intervention and education strategies for prediabetes and T2D. Furthermore, beyond the important nutritional message these findings convey, this evidence deserves further attention because the a posteriori lifestyle pattern assessment approach that was followed here reflects the true habits of the participants and not the level of adherence to an a priori-defined dietary model.

## Figures and Tables

**Table 1:** Distribution of study participants’ characteristics for the total sample and by country category.

	Total (*n* = 2759)	High-Income Countries (Belgium–Finland) (*n* = 806)	High-Income Countries under Austerity Measures (Greece–Spain) (*n* = 1280)	Low–Middle-Income Countries (Bulgaria–Hungary) (*n* = 673)	p *
	Median (IQR, 25th–75th Percentile) or (%)	Median (IQR, 25th–75th Percentile) or (%)	Median (IQR, 25th–75th Percentile) or (%)	Median (IQR, 25th–75th Percentile) or (%)	
Age (years)	41 (38–45)	39 (36–44)	43 (39–46)	39 (36–44)	**<0.001**
Sex					**<0.001**
*Male (%)*	33.7	34.1	37.7	25.6	
*Female (%)*	66.3	65.9	62.3	74.4	
Education					**<0.001**
*≤12 years (%)*	25.6	17.1	29	27.9	
*>12 years (%)*	74.4	82.9	71	72.1	
Smoking					**<0.001**
*Never smokers (%)*	45.8	56.2	43.6	38.7	
*Former smokers (%)*	28.6	32.7	28.1	25	
*Current smokers (%)*	25.6	11.1	28.3	36.3	
Existence of prediabetes					**<0.001**
*Normal (%)*	76	70.9	73.9	86.2	
*Prediabetes (%)*	24	29.1	26.1	13.8	
Body mass index (kg/m^2^)	28 (25–32)	28 (25–32)	29 (25–32)	27 (23–31)	**<0.001**
Waist circumference (cm)	95 (84–104)	95 (85–104)	96 (87–106)	90 (78–102)	**<0.001**
Fasting plasma glucose (mg/dL)	93 (86–100)	93 (86–101)	94 (88–100)	88 (83–96)	**<0.001**
Breakfast consumption (days/week)	4 (2–4)	4 (4)	4 (3–4)	3 (2–4)	**<0.001**
Sitting hours (hours/day)	5 (2.5–8)	5 (3–8)	4 (2–8)	5 (2.5–7)	**<0.001**

The reported *p*-values were calculated using the chi-square test or the Kruskal–Wallis test. * *p*-values indicate the significance of the differences among country categories. In bold, statistically significant *p*-values at 5%.

**Table 2:** Score coefficients * (loadings) derived from factor (principal component) analysis regarding lifestyle factors in the study participants.

	*Factor 1*	*Factor 2*
Fruits and berries	**0.744 ** ^†^	0.198
Vegetables	**0.672 ** ^†^	0.232
Legumes	0.236	0.081
Salty snacks	**−0.309 ** ^†^	**0.674 ** ^†^
Sweet snacks	−0.107	**0.724 ** ^†^
Soft drinks with sugar	**−0.443 ** ^†^	**0.373 ** ^†^
Juice with sugar	−0.181	**0.359 ** ^†^
Nuts and seeds	**0.586** ^†^	0.213
Alcohol	0.089	0.027
Breakfast consumption	**0.405 ** ^†^	0.193
Sedentary behavior	−0.039	**0.330 ** ^†^
Explained variation, %	17.4%	14%

* Score coefficients are similar to the correlation coefficients. Higher absolute values indicate that the lifestyle variable is correlated with the respective component. ^†^ In bold, loadings > ǀ0.3ǀ. Description of the components: component 1: breakfast consumption, high consumption of fruits and berries, vegetables and nuts and seeds, and low consumption of salty snacks and soft drinks with sugar. component 2: high consumption of salty and sweet snacks, soft drinks with sugar and juice with sugar and sedentary behavior.

**Table 3:** The association of the derived lifestyle patterns with the prevalence of prediabetes. Results are presented as odds ratios and 95% CIs.

	*Model 1*	*Model 2*	*Model 3*
*Component 1: Breakfast consumption, high consumption of fruits and berries, vegetables and nuts and seeds, and low consumption of salty snacks and soft drinks with sugar*	0.99 (0.88–1.14)	0.99 (0.87–1.13)	0.99 (0.98–1.30)
*Component 2: High consumption of salty and sweet snacks, soft drinks with sugar and juice with sugar and sedentary behavior*	**1.04 (1.02–1.06)**	**1.03 (1.02–1.05)**	**1.02 (1.01–1.04)**

All odds ratios and their corresponding 95% confidence intervals were calculated by performing multiple logistic regressions. *Note*: Bold indicates statistical significance (*P*_trend_ < 0.05). Model 1: adjusted for age and sex. Model 2: adjusted for age, sex, education and smoking. Model 3: adjusted for age, sex, education, smoking and waist circumference. Abbreviations: CI, confidence interval.

## Data Availability

The data presented in this study are available on request from the corresponding author. The data are not publicly available due to privacy. **Acknowledgments**: The authors would like to thank the members of the Feel4Diabetes Study **Group: Coordinator**: Yannis Manios; **Steering Committee**: Yannis Manios, Greet Cardon, Jaana Lindstrom, Peter Schwarz, Konstantinos Makrilakis, Lieven Annemans and Winne Ko; **Harokopio University (Greece)**: Yannis Manios, Kalliopi Karatzi, Odysseas Androutsos, George Moschonis, Spyridon Kanellakis, Christina Mavrogianni, Konstantina Tsoutsoulopoulou, Christina Katsarou, Eva Karaglani, Irini Qira, Efstathios Skoufas, Konstantina Maragkopoulou, Antigone Tsiafitsa, Irini Sotiropoulou, Michalis Tsolakos, Effie Argyri, Mary Nikolaou, Eleni-Anna Vampouli, Christina Filippou, Kyriaki Apergi, Amalia Filippou, Katerina Gatsiou and Efstratios Dimitriadis; **Finnish Institute for Health and Welfare (Finland)**: Jaana Lindström, Tiina Laatikainen, Katja Wikström, Jemina Kivelä, Päivi Valve, Esko Levälahti, Eeva Virtanen, Tiina Pennanen, Seija Olli and Karoliina Nelimarkka; **Ghent University (Belgium), Department of Movement and Sports Sciences**: Greet Cardon, Vicky Van Stappen and Nele Huys; **Ghent University (Belgium), Department of Public Health**: Lieven Annemans and Ruben Willems; **Ghent University (Belgium), Department of Endocrinology and Metabolic Diseases**: Samyah Shadid; **Technische Universität Dresden (Germany)**: Peter Schwarz and Patrick Timpel; **University of Athens (Greece)**: Konstantinos Makrilakis, Stavros Liatis, George Dafoulas, Christina-Paulina Lambrinou and Angeliki Giannopoulou; **International Diabetes Federation European Region (Belgium)**: Winne Ko and Ernest Karuranga; **Universidad De Zaragoza (Spain)**: Luis Moreno, Fernando Civeira, Gloria Bueno, Pilar De Miguel- Etayo, Esther Ma Gonzalez-Gil, María L. Miguel-Berges, Natalia Gimenez-Legarre; Paloma Flores-Barrantes, Alelí M. Ayala-Marín, Miguel Seral-Cortes, Lucia Baila-Rueda, Ana Cenarro, Estíbaliz Jarauta and Rocío Mateo-Gallego; **Medical University of Varna (Bulgaria)**: Violeta Iotova, Tsvetalina Tankova, Natalia Usheva, Kaloyan Tsochev, Nevena Chakarova, Sonya Galcheva, Rumyana Dimova, Yana Bocheva, Zhaneta Radkova, Vanya Marinova, Yuliya Bazdarska and Tanya Stefanova; **University of Debrecen (Hungary)**: Imre Rurik, Timea Ungvari, Zoltán Jancsó, Anna Nánási, László Kolozsvári, Csilla Semánova, Éva Bíró, Emese Antal and Sándorné Radó; and **Extensive Life Oy (Finland)**: Remberto Martinez and Marcos Tong.
